# Aquaculture and biodiversity‐ecosystem functioning: A path to lay the foundations of ecosystem‐based approach

**DOI:** 10.1002/eap.70334

**Published:** 2026-11-01

**Authors:** Gianluca Sarà, Yun‐Wei Dong, Maria Cristina Mangano

**Affiliations:** ^1^ Department of Earth and Marine Science (DiSTeM) University of Palermo Palermo Italy; ^2^ NBFC, National Biodiversity Future Center Palermo Italy; ^3^ Key Laboratory of Mariculture Fisheries College, Ocean University of China Qingdao China; ^4^ Dipartimento di Ecologia Marina Integrata, Sicily Marine Center SZN Stazione Zoologica Anton Dohrn Palermo Italy

**Keywords:** aquaculture, biodiversity‐ecosystem functioning, effect size, fouling communities, Mediterranean Sea, organic enrichment, Price equation

## Abstract

Marine aquaculture generates localized organic enrichment that alters benthic community composition and ecosystem functioning, particularly in nutrient‐poor systems such as the Mediterranean Sea. Current monitoring relies predominantly on abiotic variables (e.g., dissolved oxygen, nutrient loads), failing to capture the biological mechanisms through which biodiversity change affects functional outcomes. Biodiversity‐ecosystem functioning (BEF) theory links community composition to ecosystem processes but has rarely been operationalized in aquaculture contexts. Here we applied the extended Price equation to a historical dataset of hard‐substrate fouling communities–sessile filter‐feeding assemblages dominated by barnacles, bivalves and gastropods–along a gradient of organic enrichment from offshore fish cages in the Gulf of Castellammare (NW Sicily). Exploiting a hierarchical sampling design (six baseline plots × three post‐loss plots per site), we obtained 18 replicated decompositions at each of four downstream sites, enabling formal inference on five additive components of functional change. Total community biomass (ash‐free dry mass) increased significantly at all four sites relative to the upstream baseline (overall Cohen's *d* = 0.82). The species richness effect due to gain (SRE_G_) was the largest positive component (*d* = 2.59), reflecting consistent species additions at enriched sites. However, these colonizers contributed less biomass per species than the community average (SCE_G_ strongly negative, *d* = −1.76), indicating that gains were numerically important but functionally dilute. The context‐dependent effect (CDE), capturing enhanced performance of persistent filter‐feeders under organic subsidies, was the second largest driver (*d* = 0.89), significantly positive at three of four sites. The species richness effect due to loss (SRE_L_) was consistently negative but smaller in magnitude. Among environmental correlates, only the association between SCE_L_ and total suspended matter was statistically supported (bootstrap 95% CI excluding zero), while correlations between abiotic variables and total functional change were weak–highlighting the limited capacity of water‐quality monitoring to predict biodiversity‐function outcomes. This study demonstrates that the extended Price equation, applied to replicated field data, can formally partition the functional consequences of biodiversity change in aquaculture‐impacted communities. The approach provides a practical, management‐relevant diagnostic tool that complements abiotic monitoring and supports the Ecosystem Approach to Aquaculture.

## INTRODUCTION

Aquaculture has become a cornerstone of global food security (FAO, 2024), yet its expansion in coastal waters generates localized organic enrichment that cascades through benthic ecosystems, altering community composition, trophic structure and ecosystem functioning (Keeley et al., 2019; Sarà, 2007; Sarà et al., 2007). In oligotrophic systems such as the Mediterranean Sea, where baseline nutrient supply is naturally low, even moderate organic subsidies from fish cages can trigger disproportionately large shifts in the structure of hard‐substrate fouling communities–assemblages of sessile filter‐feeders (barnacles, bivalves, tube‐dwelling polychaetes) and associated mobile fauna that colonize submerged artificial and natural surfaces (Modica et al., 2006; Sarà et al., 2004). Because fouling communities are directly coupled to the water column through suspension feeding, their biomass production responds rapidly to changes in particulate food supply, making them sensitive integrators of organic enrichment gradients (Fischer et al., 2001; Montalto et al., 2020). Along such gradients, the community response is rarely a simple increase or decrease in total biomass. Species are simultaneously gained and lost; resident species change their performance, and the functional identity of colonizers may differ from that of the species they replace. Understanding these compositional and functional rearrangements, not merely their net outcome, is essential for ecologically meaningful impact assessment.

The biodiversity‐ecosystem functioning (BEF) research paradigm provides the conceptual foundation for linking such compositional changes to their functional consequences. Over the past two decades, BEF theory has demonstrated that biological diversity sustains key ecosystem processes including biomass production, nutrient cycling, decomposition and resilience to perturbation, across terrestrial, freshwater and marine systems (Cardinale et al., 2012; Loreau et al., 2022; Loreau & Hector, 2001; Woodward, 2009). Crucially, the field has moved beyond species richness as a sole predictor, recognizing that species identity, abundance, dominance structure, and context‐dependent performance are equally important in shaping ecosystem functioning (Chen et al., 2025; Gonzalez et al., 2020). However, despite its analytical maturity, BEF research remains partially confined to controlled experimental settings and has rarely been applied to real‐world marine systems subject to anthropogenic disturbance (Dee et al., 2023; Isbell et al., 2017; Mora et al., 2011). In particular, its operational integration into the management of marine food production systems–where the Ecosystem Approach to Aquaculture (EAA) has been advocated for over a decade (Soto et al., 2007) and more recently reinforced by the UNEP/MAP COP23 Portorož Declaration (UNEP/MAP, 2023a, 2023b; UNEP, 2023)–has remained largely aspirational. A key obstacle to applying BEF theory in the field is the need for analytical tools capable of handling the complexity of real communities, where species are both lost and gained and where the functional contribution of persisting species may shift with environmental context. The extended Price equation (Fox, 2006; Fox & Kerr, 2012) addresses this challenge by decomposing the total difference in ecosystem function between any two communities into five additive components: the functional effects of species loss and gain (both as random expectation and as deviation from it) and the context‐dependent change in performance of shared species. Unlike classical BEF partitions (e.g., Loreau & Hector, 2001), this framework does not require nested communities and is therefore directly applicable to observational data along disturbance gradients. Price equation has been successfully deployed in a range of ecological contexts, from grassland biodiversity experiments to large‐scale observational comparisons (Bannar‐Martin et al., 2017; Genung et al., 2020; Ladouceur et al., 2022; Lefcheck et al., 2021). Yet, to our knowledge, it has not been applied to marine fouling communities subject to aquaculture‐derived enrichment–a system where simultaneous species turnover and context‐dependent performance shifts are expected along organic loading gradients.

From a management perspective, linking biodiversity change to functional outcomes through formal partitioning of biodiversity‐function relationships has direct relevance for the development of ecosystem‐based indicators. Current aquaculture monitoring relies predominantly on abiotic variables such as, for instance, oxygen levels, nutrient concentrations, and sedimentation rates (e.g., Barrett et al., 2019; Brugère et al., 2018; Grebe et al., 2019; Sarà, 2007), which capture environmental state but not the biological processes that ultimately sustain ecosystem services. Biodiversity‐informed diagnostics, particularly those capable of detecting compensatory dynamics, functional redundancy, or threshold responses, could complement existing monitoring tools and support the transition toward the adaptive, ecosystem‐informed management called for by international frameworks including the EU Biodiversity Strategy 2030 and the UN Decade on Ecosystem Restoration (Suding & Hobbs, 2009).

Here, we apply the extended Price equation to a historical dataset characterizing hard‐substrate fouling communities along an organic enrichment gradient from offshore fish cages in the Mediterranean Sea (Sarà et al., 2007). By exploiting the hierarchical sampling design of the original study, we obtained replicated, plot‐level estimates of each Price component, enabling formal statistical inference (mean ± SE, one‐sample *t* tests, Cohen's *d*)–an advance over single‐value decompositions typically reported in the literature. We pursue three objectives: (1) to partition the BEF relationship along the enrichment gradient into its additive components (species loss, species gain, compositional change, and context dependence); (2) to explore associations between these components and trophodynamic proxies of organic enrichment (chlorophyll‐*a*, particulate organic matter [POM], and total suspended matter [TSM]); and (3) to evaluate the extended Price equation as a practical, management‐relevant diagnostic tool for aquaculture impact assessment, contributing to the operationalization of the EAA.

## MATERIALS AND METHODS

### Study system and dataset

This study builds on the sampling campaign originally described by Sarà et al. (2007), which reported site‐level aggregates of hard‐substrate fouling community structure and biomass along a gradient of organic enrichment from offshore fish cages in the Gulf of Castellammare (NW Sicily, Southern Tyrrhenian Sea; Figure 1). Here, we exploit the full hierarchical dataset (plots and within‐plot replicates) from that campaign, which has not been previously published in disaggregated form. The complete replicated matrix is provided in Appendix S1: Table S1. Fouling communities colonizing artificial substrates are dominated by sessile filter‐feeding organisms–primarily barnacles (*Balanus perforatus*, *Chthamalus stellatus*), bivalves (*Mytilus galloprovincialis*, *Ostrea edulis*, *Modiolus minimus*), and gastropods–whose biomass production is tightly linked to particulate food supply, making them sensitive indicators of organic enrichment gradients (Fischer et al., 2001; Montalto et al., 2020). Artificial settlement units were deployed at six sites and retrieved after 1 year of submersion, allowing the accumulation of representative assemblages under different levels of aquaculture‐derived organic enrichment. Two sites were positioned upstream of the fish cages and served as the undisturbed baseline (CTRL, two sites, three plots each); four additional sites were located at increasing distances downstream from the cages, reflecting a gradient of decreasing organic impact (Figure 1). One downstream site (site 3 in the original numbering of Sarà et al., 2007) was excluded from the analysis because multiple independent lines of evidence indicated that it did not effectively receive aquaculture‐derived organic enrichment. Specifically, its community composition was virtually indistinguishable from the baseline (Bray–Curtis dissimilarity = 0.20, compared with 0.43–0.70 for all other post‐loss sites), its POM concentration was lower than the upstream control itself (467 vs. 516 μg L^−1^), and it was the only site exhibiting a negative change in total ecosystem function (Δ*T* = −3.1‐g ash‐free dry mass [AFDM]). Together, these observations suggest that local hydrodynamic conditions prevented effective organic loading at this site, which therefore could not serve as a meaningful post‐loss comparison. The final design thus comprised two baseline and four post‐loss sites–originally numbered Post‐loss 1, 2, 4, and 5 in Sarà et al. (2007), renumbered here as Post‐loss 1–4 for clarity–yielding a total of 72 replicated Price equation decompositions (six CTRL plots × three IMP plots × four sites). At each site, three replicate plots were sampled; within each plot, three replicates (20 × 20 cm) were collected. For each replicate, all organisms were identified to species and counted (abundance, N); AFDM (in grams) was determined by loss on ignition and served as the proxy for ecosystem functioning throughout this study. The dataset comprises 14 taxa (Table 1), spanning barnacles, bivalves, gastropods, and echinoderms. Plot‐level values were computed as the mean AFDM per species across the three replicates within each plot, yielding six baseline plots (two CTRL sites × three plots) and three plots per post‐loss site (Figure 2).

**FIGURE 1:**
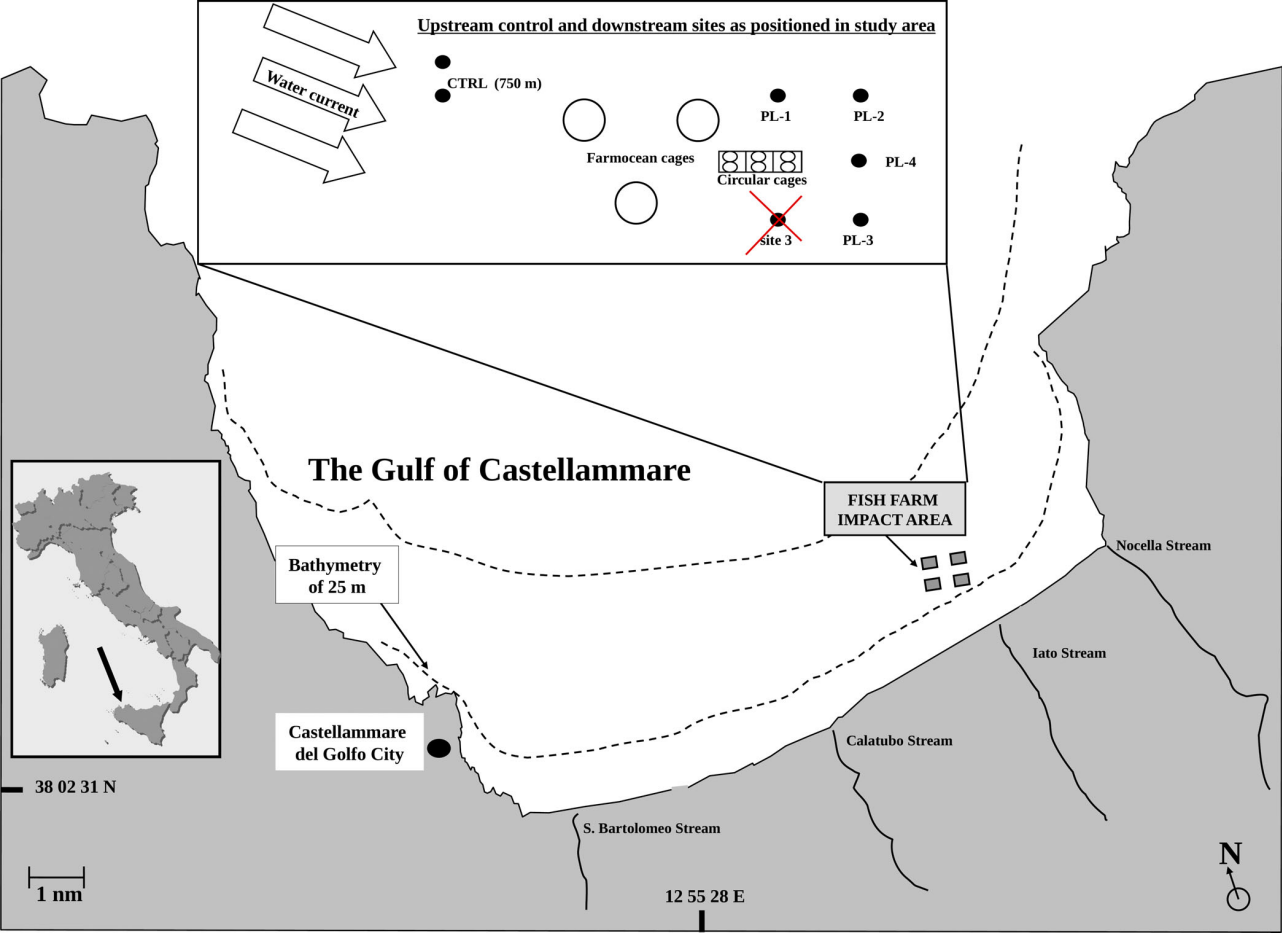
Study area in the Gulf of Castellammare (NW Sicily, Southern Tyrrhenian Sea) showing the spatial arrangement of sampling sites along the organic enrichment gradient from offshore fish cages. The baseline (CTRL; *n* = 2) was located upstream of the aquaculture facility; the five post‐loss sites originally sampled by Sarà et al. (2007) were positioned progressively downstream. Site 3 (original numbering) was excluded from the present analysis; the four retained sites were renumbered post‐loss sites (PL‐1 to PL‐4) (see *Materials and Methods*) (modified from Sarà et al. [2007]).

**TABLE 1:** Fouling species recorded in the study area (Gulf of Castellammare, NW Sicily).

Species	Higher taxon	Trophic group	CTRL	PL‐1	PL‐2	PL‐3	PL‐4	Notes
*Balanus perforatus* (Bruguière, 1789)	Cirripedia	Suspension feeder	●	●	●	●	●	Dominant barnacle; main biomass contributor across all sites
*Chthamalus stellatus* (Poli, 1791)	Cirripedia	Suspension feeder	●	●	…	●	●	Co‐dominant barnacle at baseline; declines at enriched sites
*Balanus trigonus* Darwin, 1854	Cirripedia	Suspension feeder	…	…	…	…	●	Only at most distant downstream site (PL‐4)
*Pachygrapsus marmoratus* (Fabricius, 1787)	Decapoda	Omnivore	●	●	●	…	●	Shore crab; low biomass throughout
*Eriphia verrucosa* (Forskål, 1775)	Decapoda	Carnivore	●	…	…	●	●	Predatory crab; sporadic
*Acanthonyx lunulatus* (Risso, 1816)	Decapoda	Herbivore/omnivore	●	●	●	●	●	Small decorator crab; negligible biomass
*Ostrea edulis* Linnaeus, 1758	Bivalvia	Suspension feeder	●	…	●	●	●	European flat oyster; variable biomass
*Mytilaster minimus* (Poli, 1795)	Bivalvia	Suspension feeder	●	●	●	●	●	Small mussel; abundance increases sharply with enrichment
*Mytilus galloprovincialis* Lamarck, 1819	Bivalvia	Suspension feeder	●	●	●	●	●	Mediterranean mussel; present at all sites
*Brachidontes pharaonis* (Fischer P., 1870)	Bivalvia	Suspension feeder	…	●	●	●	●	Lessepsian migrant; absent at baseline, gains under enrichment
*Arca noae* Linnaeus, 1758	Bivalvia	Suspension feeder	●	…	…	…	…	Noah's ark shell; only at baseline
*Patella coerulea* Linnaeus, 1758	Gastropoda	Grazer	●	●	●	●	…	Limpet; low biomass
*Stramonita haemastoma* (Linnaeus, 1767)	Gastropoda	Predator	…	●	●	●	…	Predatory whelk; sporadic at enriched sites
*Arbacia lixula* (Linnaeus, 1758)	Echinoidea	Grazer	…	●	●	●	●	Black sea urchin; absent at baseline, colonizes downstream

*Note*: Occurrence (●) at the baseline (CTRL) and four retained post‐loss sites (renumbered PL‐1 to PL‐4; the site excluded from analysis was site 3 in the original numbering of Sarà et al., 2007, see *Materials and Methods*) based on mean AFDM >0 across plots. Trophic groups follow the classification in Sarà et al. (2007); AFDM, ash‐free dry mass. The complete species × plot data matrix is provided in Appendix S1: Table S1. *Stramonita haemastoma* was listed as *Thais haemastoma* in Sarà et al. (2007); nomenclature updated following WoRMS (WoRMS Editorial Board, 2026). Polychaeta (present but not identified to species) is excluded.

**FIGURE 2:**
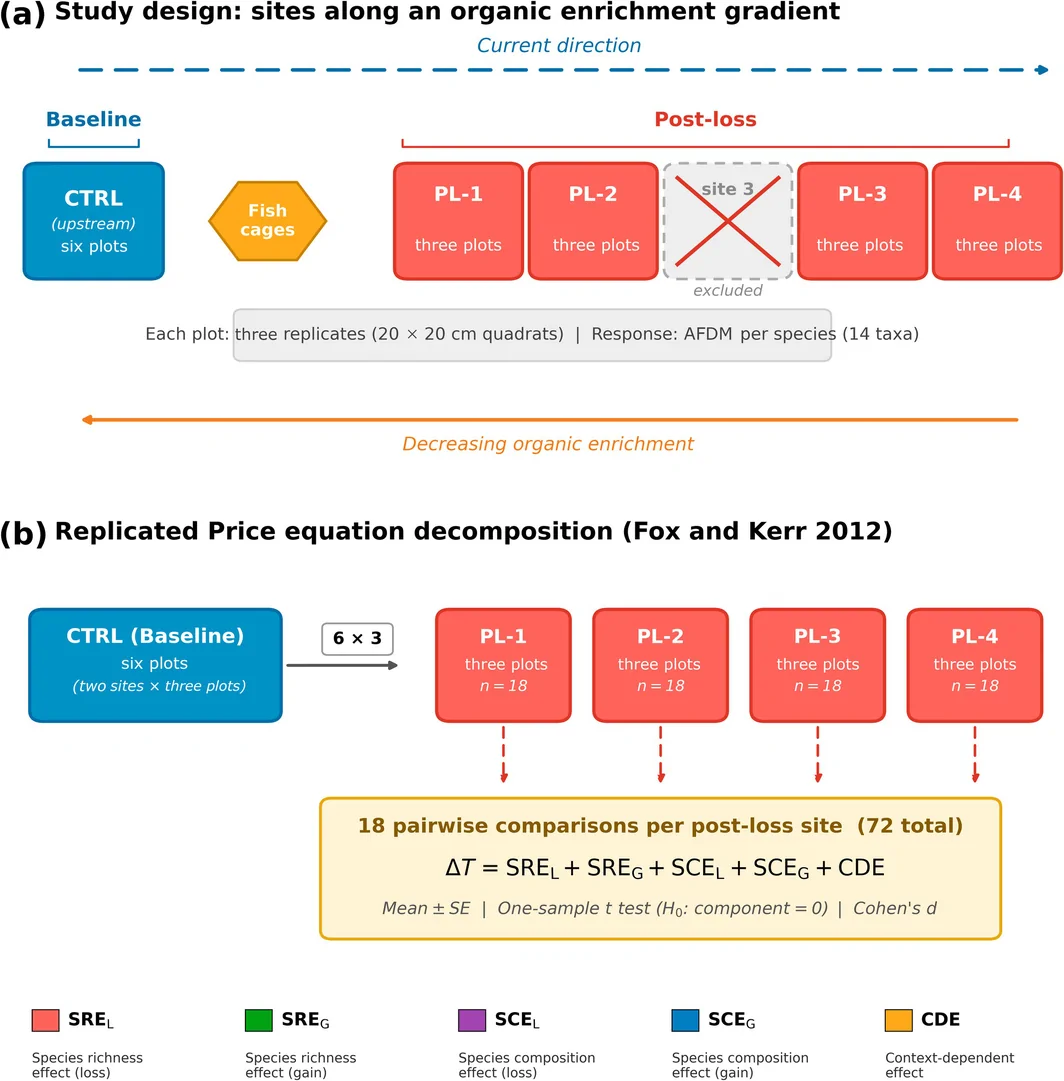
Conceptual overview of the study design and analytical framework. (a) Spatial layout of sites along the organic enrichment gradient downstream of the fish cages, showing the upstream baseline (CTRL, two sites, six plots) and the four retained post‐loss sites (PL‐1 to PL‐4; three plots each; site 3 in the original numbering of Sarà et al., 2007 was excluded, see *Materials and Methods*). Each plot contained three replicate 20 × 20 cm quadrats; the response variable was ash‐free dry mass (AFDM) per species for each of 14 taxa. (b) Replicated Price equation decomposition: Each of the six baseline plots was compared pairwise with each of the three post‐loss plots, yielding 18 comparisons per site (72 total) (Fox & Kerr, 2012). Each comparison produced the five‐component partition Δ*T* = SRE_L_ + SRE_G_ + SCE_L_ + SCE_G_ + CDE. Results were evaluated as mean ± SE with one‐sample *t* tests and Cohen's *d*.

Additionally, we utilized unpublished trophodynamic data from a project report (Sarà et al., 2004), including measurements taken at each site upstream and downstream of the aquaculture facility. Trophic conditions were characterized by three proxies: (1) suspended chlorophyll‐*a* (CHL‐*a*, μg L^−1^), representing autotrophic primary biomass, measured from Niskin‐bottle water samples following Sarà et al. (2006) and the method of Lorenzen and Jeffrey (1982); (2) POM (μg L^−1^), calculated as the sum of lipids, proteins, and carbohydrates embedded in the seston, quantified by standard biochemical methods (Sarà & Mazzola, 1997); proteins: Lowry et al. (1951), as modified by Hartree (1972) and Rice (1982); carbohydrates: Gerchakov and Hatcher (1972); lipids: Bligh and Dyer (1959) and Marsh and Weinstein (1966); and (3) TSM (mg L^−1^), representing the gross bulk of suspended material (organic and inorganic), measured by loss on ignition (Strickland & Parsons, 1972). CHL‐*a* and POM samples were pre‐filtered through a 200‐μm mesh to exclude macrozooplankton; TSM samples were unfiltered. These variables are used here solely to explore their association with the Price equation components and are not intended to characterize the full trophodynamic regime of the study area, which is beyond the scope of this study.

### The extended Price equation

To partition observed changes in ecosystem function between the baseline and each post‐loss community, we applied the extended Price equation framework (Fox & Kerr, 2012), which generalizes the earlier formulation of Fox (2006) to communities that are not nested (i.e., where species are both lost and gained). The framework decomposes the total difference in function (Δ*T*) between a baseline community and a comparison community into five additive components: the species richness effect due to loss (SRE_L_), the species richness effect due to gain (SRE_G_), the species composition effect due to loss (SCE_L_), the species composition effect due to gain (SCE_G_) and the context‐dependent effect (CDE), such that

$$\Delta T={\mathrm{SRE}}_{L}+{\mathrm{SRE}}_{G}+{\mathrm{SCE}}_{L}+{\mathrm{SCE}}_{G}+\mathrm{CDE}$$

SRE_L_ and SRE_G_ quantify the functional change expected from random loss or gain of species, respectively; SCE_L_ and SCE_G_ capture deviations from random expectation arising when the species lost or gained are functionally nonrandom. CDE measures changes in the functional contribution of species shared between both communities. In our application, the baseline community is the upstream, undisturbed controls (CTRL: two sites, six plots), and the comparison community is each post‐loss site positioned downstream along the enrichment gradient (three plots per site). AFDM is the functional variable. Detailed derivations and worked examples are provided in Fox (2006), Fox and Kerr (2012), and Genung et al. (2020) while the method has been applied in a range of BEF contexts including experimental and observational systems (Bannar‐Martin et al., 2017; Ladouceur et al., 2022).

### Statistical analysis

We exploited the hierarchical sampling design to obtain replicated estimates of the Price equation components. For each post‐loss site, every one of the six CTRL plots was compared pairwise with every one of the three post‐loss plots, yielding 18 independent decompositions per post‐loss site and 72 decompositions overall (Figure 2a,b). In each pairwise comparison, species present in the CTRL plot but absent from the post‐loss plot were classified as lost; species absent from the CTRL plot but present in the post‐loss plot were classified as gained. Species present in both were classified as shared. The five Price components and Δ*T* were then computed following Fox and Kerr (2012). For each Price component at each post‐loss site, we report the mean ± SE across the 18 pairwise comparisons. Whether each component differed significantly from zero was assessed by one‐sample *t* tests (H_0_: μ = 0, two‐tailed; α = 0.05). This approach treats each CTRL‐post‐loss plot pair as an independent replicate of the Price decomposition, directly addressing the need for statistical inference and uncertainty estimation around point estimates. We also computed an overall estimate by pooling all 72 comparisons across the four post‐loss sites.

### Environmental correlations

To explore potential associations between Price equation components and trophodynamic conditions, we calculated Pearson correlation coefficients between site‐level mean values of each component and the three environmental variables (TSM, CHL‐*a*, POM). Because environmental variables were measured at the site level while Price components were estimated at the plot level, correlations were computed on site means (*n* = 4) to avoid pseudoreplication. Given the small sample size, uncertainty was assessed through nonparametric bootstrap 95% CIs (10,000 iterations, resampling sites with replacement) rather than parametric *p*‐values. A correlation was considered noteworthy when its bootstrap CI excluded zero. This two‐tiered approach–plot‐level analysis for the Price decomposition, site‐level analysis for environmental correlations–is consistent with the hierarchical structure of the data (Hurlbert, 1984).

### Effect size estimation

To quantify the standardized magnitude of each component's contribution to functional change, we computed Cohen's *d* for each Price component at each post‐loss site as

$$d=\bar{x}/\mathrm{SD}$$

where *x̄* is the mean value of the component across the 18 pairwise comparisons and SD is the corresponding standard deviation. This formulation is equivalent to a one‐sample Cohen's *d* measuring the distance of the distribution from zero in standardized units. Effect sizes were interpreted following conventional thresholds: |*d*| ≈0.2 (small), ≈0.5 (medium) and ≥0.8 (large) (Cohen, 1988). Approximate 95% CIs for *d* were obtained as *d* ± 1.96 × √(1/*n* + *d* ^2^/2*n*), where *n* = 18 (Hedges & Olkin, 1985). This effect size analysis complements the absolute values reported in Table 2 by providing a standardized, scale‐independent comparison across components and sites. All analyses were performed in Python 3.12 using NumPy, SciPy, and Matplotlib.

**TABLE 2:** One‐sample *t* tests (H₀: Component = 0) for the five Price equation components and total functional change (Δ *T*) at each post‐loss site.

	PL‐1 *t*	PL‐1 *p*	PL‐2 *t*	PL‐2 *p*	PL‐3 *t*	PL‐3 *p*	PL‐4 *t*	PL‐4 *p*
SRE_L_	−4.649	<0.001***	−5.403	<0.001***	−5.127	<0.001***	−3.290	0.0043**
SRE_G_	12.245	<0.001***	13.581	<0.001***	15.799	<0.001***	9.977	<0.001***
SCE_L_	−0.801	0.4343	−0.551	0.5889	2.667	0.0163*	3.793	0.0015**
SCE_G_	−11.418	<0.001***	−9.017	<0.001***	−8.783	<0.001***	−5.996	<0.001***
CDE	7.454	<0.001***	7.205	<0.001***	1.654	0.1165	3.073	0.0069**
Δ*T*	2.511	0.0224*	3.668	0.0019**	3.058	0.0071**	4.701	<0.001***

*Note*: Values are *t* statistics with corresponding *p*‐values based on 18 pairwise CTRL‐post‐loss comparisons per site. Significance: *p < 0.05, **p < 0.01, ***p < 0.001.

## RESULTS

### Component‐specific contributions to ecosystem functional change

Application of the extended Price equation (Fox & Kerr, 2012) partitioned changes in total ecosystem function (Δ*T*, measured as AFDM biomass) between the baseline community (CTRL, upstream) and four post‐loss sites positioned downstream along a gradient of aquaculture‐derived organic enrichment (Figure 3). Values reported below are means ± SE; significance was assessed by one‐sample *t* tests (H₀: component = 0).

**FIGURE 3:**
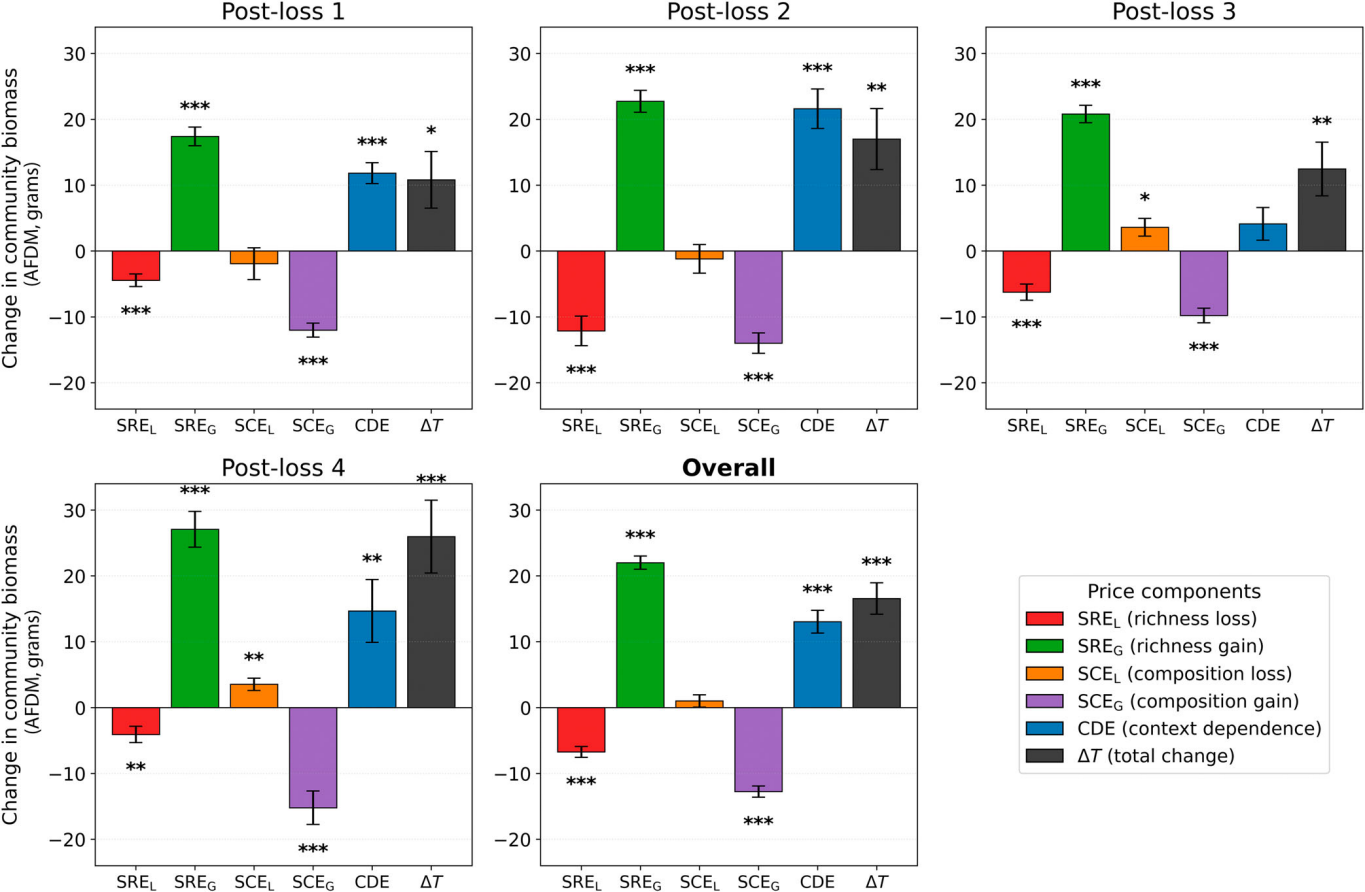
Component‐specific contributions to the total difference in ecosystem functioning (Δ*T*) across four post‐disturbance (post‐loss) sites along a gradient of organic enrichment from offshore aquaculture. Bars represent individual components derived from the extended Price equation (Fox & Kerr, 2012): species richness effect due to loss (SRE_L_, red), species richness effect due to gain (SRE_G_, green), species composition effect due to loss (SCE_L_, orange), species composition effect due to gain (SCE_G_, purple), context dependence effect (CDE, blue), and total functional change (Δ*T*, dark gray). Error bars represent ±1 SE from 18 pairwise CTRL–IMP comparisons per site. The rightmost panel pools all 72 comparisons. Significance: *p < 0.05, **p < 0.01, *** *p* < 0.001 (one‐sample *t* test, H_0_: Component = 0).

The net functional change (Δ*T*) was significantly positive at all four post‐loss sites (Figure 3; Appendix S1: Table S2), indicating that fouling community biomass was consistently higher at impacted sites relative to the upstream control. Δ*T* ranged from +10.8 ± 4.3 g AFDM at Post‐loss 1 (*t* = 2.51, *p* = 0.022) to +26.0 ± 5.5 g at Post‐loss 4 (*t* = 4.70, *p* < 0.001). The overall effect pooled across 72 comparisons was +16.5 ± 2.4 g (*t* = 6.93, *p* < 0.001; Cohen's *d* = 0.82), confirming a large, robust positive effect of aquaculture‐derived enrichment on community‐level biomass production.

The SRE_G_ was the largest positive component, consistently and highly significantly different from zero across all sites (all *p* < 0.001; Appendix S1: Table S2). SRE_G_ ranged from +17.4 ± 1.4 g at Post‐loss 1 to +27.1 ± 2.7 g at Post‐loss 4, with an overall mean of +22.0 ± 1.0 g (Cohen's *d* = 2.59), reflecting very large standardized effects. This indicates that species colonizing the enriched sites contributed substantial biomass simply through their presence. Conversely, the SRE_L_ was uniformly negative and significant at all sites (*p* ≤ 0.01), ranging from −4.1 ± 1.2 g at Post‐loss 4 to −12.1 ± 2.2 g at Post‐loss 2. The overall SRE_L_ was −6.7 ± 0.8 g (*d* = −0.96), representing a moderate‐to‐large negative effect. However, the magnitude of SRE_G_ consistently exceeded that of SRE_L_ at every site, resulting in a net positive contribution of species turnover to Δ*T*.

The context dependence effect (CDE), reflecting changes in biomass production by species shared between baseline and post‐loss communities, was the second most important positive driver. CDE was significantly positive at three of four sites: Post‐loss 1 (+11.8 ± 1.6 g; *p* < 0.001), Post‐loss 2 (+21.6 ± 3.0 g; *p* < 0.001), and Post‐loss 4 (+14.7 ± 4.8 g; *p* = 0.007). At Post‐loss 3, CDE was positive but not statistically distinguishable from zero (+4.1 ± 2.5 g; *p* = 0.117). Overall, CDE averaged +13.0 ± 1.7 g (*d* = 0.89; *p* < 0.001). These results indicate that persistent species generally performed better (i.e., accumulated more biomass) in the enriched post‐loss environments, consistent with enhanced trophic resource availability stimulating growth of filter‐feeding taxa.

The species composition effect due to gain (SCE_G_) was consistently and strongly negative across all sites (all *p* < 0.001), ranging from −9.8 ± 1.1 g at Post‐loss 3 to −15.2 ± 2.5 g at Post‐loss 4 (overall: −12.8 ± 0.9 g; *d* = −1.76). This indicates that species gained at the post‐loss sites contributed less biomass per species than the average species already present, suggesting that colonizers were relatively low‐performing compared to established residents.

The species composition effect due to loss (SCE_L_) showed a site‐dependent pattern. It was nonsignificant at Post‐loss 1 (−1.9 ± 2.4 g; *p* = 0.434) and Post‐loss 2 (−1.2 ± 2.2 g; *p* = 0.589), but significantly positive at Post‐loss 3 (+3.6 ± 1.3 g; *p* = 0.016) and Post‐loss 4 (+3.5 ± 0.9 g; *p* = 0.002). Positive SCE_L_ values indicate that species lost from the baseline were below‐average performers; their nonrandom loss therefore mitigated the functional decline expected from richness reduction alone.

### Environmental correlates of functional partitioning components

To explore potential environmental drivers, site‐level Pearson correlations were calculated between the mean Price components and three trophic proxies (TSM, CHL‐a, POM) measured at each post‐loss site (Table 3; Figure 4). Because environmental variables were measured at the site level while Price components were estimated at the plot level, correlations were computed on site means (*n* = 4) and uncertainty was assessed through bootstrap 95% CIs (10,000 iterations, resampling sites with replacement) rather than parametric *p*‐values to avoid pseudoreplication.

**TABLE 3:** Pearson correlations between Price components and environmental variables (site‐level correlations, *n* = 4).

	SRE_L_	SRE_G_	SCE_L_	SCE_G_	CDE	Δ*T*
CHL‐*a*	0.896 [−1.000, 1.000]	0.115 [−1.000, 1.000]	0.731 [−1.000, 1.000]	0.303 [−1.000, 1.000]	−0.799 [−1.000, 1.000]	0.134 [−1.000, 1.000]
TSM	−0.008 [−1.000, 1.000]	0.318 [−1.000, 1.000]	**0.840 [0.583, 1.000]**	0.439 [−1.000, 1.000]	−0.576 [−1.000, 1.000]	0.092 [−1.000, 1.000]
POM	−0.688 [−1.000, 1.000]	−0.158 [−1.000, 1.000]	0.096 [−1.000, 1.000]	0.534 [−1.000, 1.000]	−0.151 [−1.000, 1.000]	−0.403 [−1.000, 1.000]

*Note*: Values shown as *r* (bootstrap 95% CI). Bold indicates CI excludes zero.

Abbreviations: SCE_G_, species composition effect due to gain; SCE_L_, species composition effect due to loss; SRE_G_species richness effect due to gain; SRE_L_, species richness effect due to loss; Δ*T*, total functional change.

**FIGURE 4:**
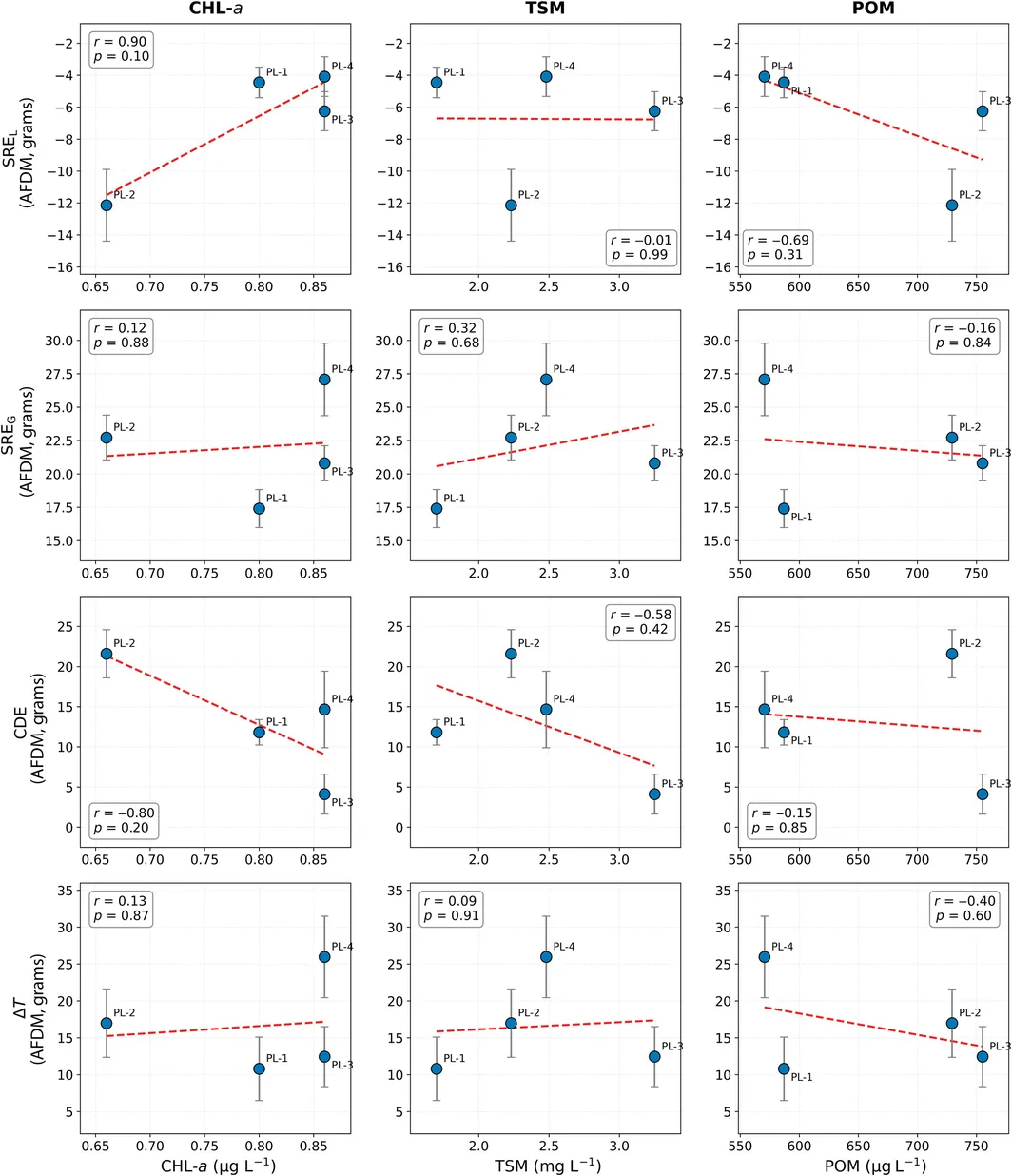
Site‐level Pearson correlations between key Price equation components (species richness effect due to loss [SRE.L], species richness effect due to gain [SRE.G], context‐dependent effect [CDE], total functional change [Δ*T*]) and environmental variables (chlorophyll‐*a* [CHL‐*a*], total suspended matter [TSM], particulate organic matter [POM]) across the four retained post‐loss sites. Each point represents a site‐level mean (± SE) of the Price component plotted against the corresponding environmental measurement. Dashed lines indicate linear regression fits. Correlation coefficients (*r*) and parametric *p*‐values are shown for each panel; bootstrap 95% CIs are reported in Table 3. CDE, context dependence effect; SCE_G_, species composition effect due to gain; SCE_L_, species composition effect due to loss; SRE_G_species richness effect due to gain; SRE_L_, species richness effect due to loss; Δ*T*, total functional change.

The only correlation whose bootstrap CI excluded zero was between SCE_L_ and TSM (*r* = 0.84; 95% CI [0.58, 1.00]), suggesting that higher suspended matter loads were associated with a greater mitigating effect of nonrandom species loss on functional decline. Several other correlations showed large point estimates but wide confidence intervals consistent with the small sample size: SRE_L_ versus CHL‐a (*r* = 0.90), CDE versus CHL‐a (*r* = −0.80), and SRE_L_ versus POM (*r* = −0.69). These effect sizes, while not formally significant with *n* = 4, suggest that chlorophyll‐*a* and suspended matter may modulate the magnitude of richness‐dependent effect and CDE along the enrichment gradient. POM showed generally weaker associations with all Price components.

### Standardized effect sizes across functional components

Cohen's *d* values (Figure 5; Appendix S1: Table S3) revealed substantial heterogeneity in the strength of ecological drivers among sites, but a consistent overall pattern. SRE_G_ exhibited the largest standardized effects at every site (*d* ranging from 2.35 to 3.72; overall *d* = 2.59), followed by SCE_G_ with consistently large negative effects (*d* from −1.41 to −2.69; overall *d* = −1.76). CDE showed larger positive effects at Post‐loss 1 and 2 (*d* = 1.76 and 1.70) but a smaller, nonsignificant effect at Post‐loss 3 (*d* = 0.39). SRE_L_ was negative with large effects at Post‐loss 1, 2, and 4 (|*d*| > 1.0) and a moderate effect at Post‐loss 4 (|*d*| = 0.78). The total functional change (Δ*T*) showed medium to large positive effects across all sites (*d* = 0.59–1.11; overall *d* = 0.82), confirming a robust, ecologically meaningful enhancement of ecosystem functioning along the enrichment gradient. The forest plot (Figure 5) visually highlights that the dominant ecological signal is the interplay between strong species gains (SRE_G_ ≫ 0) partially offset by low per‐species performance of colonizers (SCE_G_ ≪ 0), with CDE providing additional positive modulation at most sites.

**FIGURE 5:**
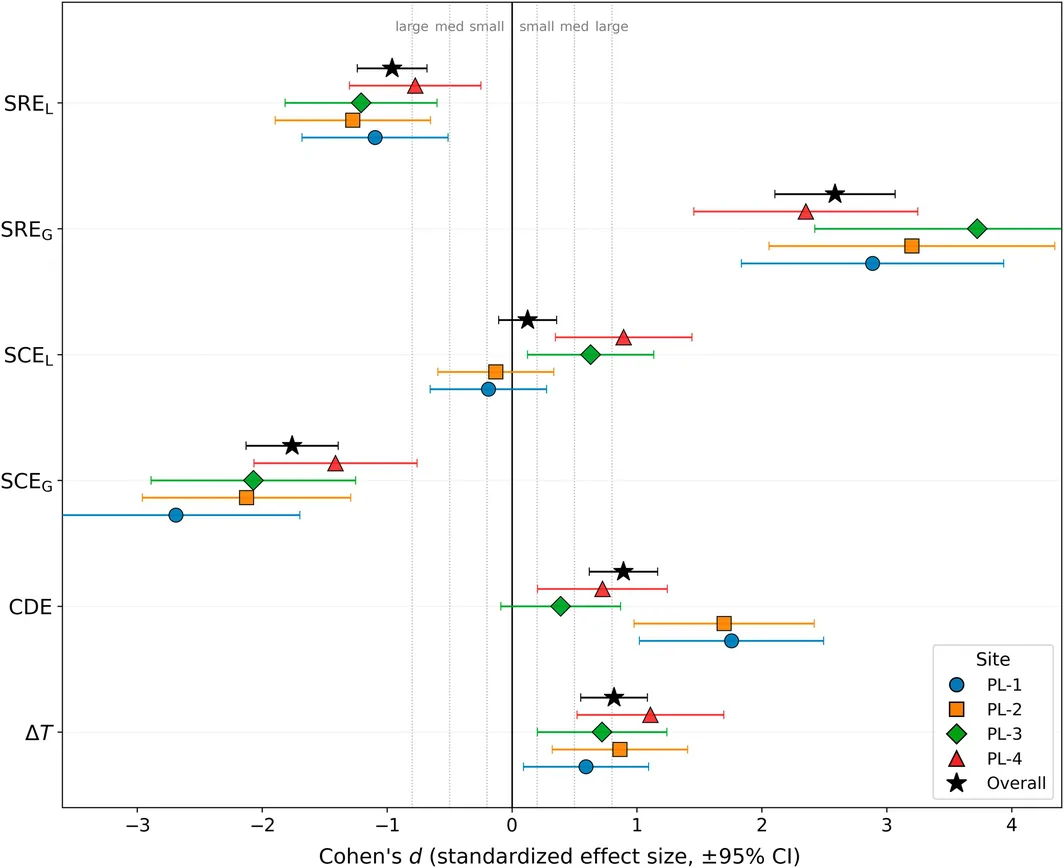
Forest plot of standardized effect sizes (Cohen's *d* ± 95% CI) for the five Price equation components at each post‐loss site and overall. Vertical dashed lines indicate conventional effect size thresholds (|*d*| = 0.2, small; 0.5, medium; 0.8, large). Different symbols represent post‐loss sites (circles: PL‐1; squares: PL‐2; diamonds: PL‐3; triangles: PL‐4) and the overall estimate (stars). Component labels appear on the left axis. SCE_G_, species composition effect due to gain; SCE_L_, species composition effect due to loss; SRE_G_species richness effect due to gain; SRE_L_, species richness effect due to loss; Δ*T*, total functional change.

## DISCUSSION

### Additive decomposition of functional change along the enrichment gradient

By applying the extended Price equation (Fox & Kerr, 2012) to replicated fouling community data across a gradient of aquaculture‐derived organic enrichment, this study achieved a formal additive decomposition of the BEF relationship that goes beyond the net change in biomass typically reported in aquaculture impact assessments. The consistent, significantly positive Δ*T* across all four post‐loss sites (Cohen's *d* = 0.59–1.11) demonstrates that organic enrichment enhanced community‐level biomass production–a finding broadly consistent with the documented stimulatory effects of aquaculture subsidies on suspension‐feeding assemblages in oligotrophic Mediterranean waters (Liao et al., 2019; Sarà, 2007; Sarà et al., 2007). However, the Price decomposition reveals that this net positive outcome masks a complex interplay of opposing processes: substantial species gains, partially offset by below‐average performance of colonizers, modulated by enhanced performance of persistent species and mitigated by the functional cost of species loss. No single component dominated uniformly, underscoring the value of the partitioning approach.

### Added value over conventional community analysis

The broad ecological patterns reported here–stable species richness, increased total biomass and a compositional shift from barnacle‐ to bivalve‐dominated assemblages under organic enrichment–were already documented in the original survey of the same system (Sarà et al., 2007). The Price decomposition does not overturn those findings but reveals three features that were inaccessible through conventional community analysis. First, it exposes an asymmetry between the numerical and functional contribution of species gained at enriched sites: colonizers such as *Arbacia lixula*, *Stramonita haemastoma*, and *Brachidontes pharaonis* were consistently present (SRE_G_ strongly positive) yet contributed individually less biomass than the community average (SCE_G_ strongly negative), a decoupling invisible to standard richness or aggregate biomass metrics. Second, it separates the contribution of species turnover from the enhanced performance of persistent species, showing that approximately half of the total functional increase (Δ*T*) was attributable to the CDE–that is, to resident filter‐feeders performing better under enriched conditions–rather than to the arrival of new species. Third, it quantifies the weak predictive capacity of routinely monitored environmental variables for the net functional outcome, a finding with direct management implications that the 2007 analysis, structured around permutational analysis of variance on species composition, was not designed to address. Collectively, these insights illustrate that the Price equation adds diagnostic resolution–the ability to identify *where* within the biodiversity‐function relationship the change occurs–to the descriptive resolution already provided by multivariate community ecology.

### Context dependence as a primary driver of functional enhancement

The CDE emerged as the second largest positive contributor to Δ*T* (overall *d* = 0.89), significantly positive at three of four sites and positive though nonsignificant at the fourth. CDE captures the change in biomass production by species shared between the baseline and comparison communities, and its consistently positive values indicate that persistent species performed better under enriched conditions. In the study system, the dominant filter‐feeders–*B. perforatus*, *Mytilaster minimus* and *M. galloprovincialis*–are directly coupled to the water column through suspension feeding and their growth responds positively to increased particulate food availability (Sarà, 2006; Sarà et al., 2004, 2008). The large positive CDE values, particularly at Post‐loss 1 and 2 (the sites closest to the cages and presumably experiencing the highest organic loading), are therefore consistent with a trophic subsidy whereby aquaculture‐derived organic matter enhances the performance of resident suspension feeders (Table 4). This finding reinforces the argument that monitoring species performance–not merely species presence–is essential for detecting functional responses to environmental change (Bosch‐Belmar et al., 2025).

**TABLE 4:** Candidate ecological mechanisms consistent with the observed direction of each Price equation component along the organic enrichment gradient.

Component	Observed pattern	Candidate mechanism(s)	Supporting evidence	Experimental test
*SRE*_*L*_	Negative at all sites	*Competitive exclusion for space*. Expanding bivalve beds (*Mytilaster*, *Mytilus*) overgrow and smother smaller barnacles (*Chthamalus*) *Sedimentation smothering*. Increased organic sedimentation clogs filtration apparatus of small cirripedes	Durr and Wahl (2004): mussels outcompete barnacles under intermediate disturbance; Sarà et al. (2007): *Chthamalus* absent at most enriched sites	Removal experiment: selectively clear bivalves from enriched plots and monitor barnacle recolonization
*SRE*_*G*_	Strongly positive at all sites	*Biogenic habitat facilitation*. Increased structural complexity from filter‐feeder growth creates microhabitats for new colonizers (*Arbacia*, *Stramonita*) *Direct trophic subsidy*. Elevated particulate food supports species unable to persist under oligotrophic baseline conditions *Invasion facilitation*. Organic enrichment lowers biotic resistance, enabling establishment of invasive *Brachidontes pharaonis* (Lessepsian migrant)	Mook (1981): fouling structural complexity drives recruitment; Sarà et al. (2013, 2018): *B. pharaonis* expanding in western Mediterranean under warming and disturbance	Deploy standardized substrates with/without preestablished bivalve beds at enriched and control sites; monitor recruitment over 6–12 months
*SCE*_*L*_	Nonsignificant (PL‐1, PL‐2); positive (PL‐3, PL‐4)	*Selective loss of minor contributors*. At distant sites, species lost were below‐average performers; their departure is functionally neutral or beneficial (*mass ratio hypothesis*, Grime, 1998) *Stochastic loss at proximal sites*. Near‐cage losses are random with respect to function, hence SCE_L_ ≈0	Avolio et al. (2019): dominant species disproportionately drive functioning; species lost at PL‐3/4 had low baseline AFDM	Selective removal of high‐ versus low‐biomass species and comparison of functional response
*SCE*_*G*_	Strongly negative at all sites	*Demographic lag*. Colonizers are recently recruited, small‐bodied individuals that have not yet reached adult biomass–an ontogenetic effect rather than intrinsic species limitation *Niche marginality*. Gained species occupy peripheral trophic niches (grazer, predator) with inherently lower biomass than dominant suspension feeders	*Arbacia* and *Stramonita* are mobile grazers/predators, not filter‐feeders; *B. pharaonis* individuals at early invasion stage are typically small (Sarà et al., 2013)	Temporal monitoring: repeat sampling after 1–2 years to distinguish demographic lag from intrinsic functional limitation
*CDE*	Positive at three of four sites; strongest near cages	*Trophic subsidy* (*direct*). Higher particulate organic matter increases filtration rates and somatic growth of persistent suspension feeders *Competitive release*. Loss of some competitors frees space and resources for remaining species, enhancing per capita performance *Size‐structure shift*. Differential survival of larger individuals under enrichment inflates mean per capita biomass without individual growth change	*Mytilus* growth positively correlated with particulate food in Mediterranean; DEB models predict increased performances under elevated food (Sarà et al., 2012)	*Additive BEF experiment* sensu Loreau and Hector (2001): monoculture versus mixture substrates deployed at control and enriched sites; individual growth rates measured in situ

*Note*: The extended Price equation identifies the direction and magnitude of each component but does not test causal mechanisms; the interpretations below represent hypotheses derived from the known biology of the study system (Sarà et al., 2007) and the broader literature on fouling community dynamics. The final column proposes experimental approaches for direct validation.

### Species gains outweigh losses, but colonizers underperform

The SRE_G_ was the single largest component of Δ*T*, with very large standardized effects at every site (overall *d* = 2.59). This reflects the consistent addition of species at post‐loss sites–notably the urchin *A. lixula*, the gastropod *S. haemastoma* and the invasive mussel *B. pharaonis*–whose presence contributed substantial aggregate biomass. However, the strongly negative SCE_G_ (overall *d* = −1.76) reveals that these colonizers contributed less biomass per species than the community average, indicating that species gains were numerically substantial but individually low‐performing relative to the community average. This pattern is ecologically coherent: Colonizing species are often smaller bodied, less established or early‐successional relative to the dominant residents they join (Genung et al., 2020) and may also occupy peripheral trophic niches–grazers and predators–with inherently lower biomass than the dominant suspension feeders (Table 4). Whether this functional dilution reflects an intrinsic species limitation or a demographic lag (recently recruited individuals that have not yet reached adult biomass) remains an open question that would require temporal monitoring to resolve.

Conversely, the SRE_L_ was consistently negative but smaller in magnitude than SRE_G_, yielding a net positive richness‐driven contribution to Δ*T*. The species composition effect due to loss (SCE_L_) was site‐dependent: nonsignificant at Post‐loss 1 and 2, but significantly positive at Post‐loss 3 and 5, indicating that at these more distant sites, the species lost tended to be below‐average performers–their nonrandom departure mitigated the expected functional decline. This aligns with the mass ratio hypothesis (Grime, 1998): When the lost species are minor contributors, their disappearance has limited functional impact, whereas the loss of dominant suspension feeders (as implied by the larger SRE_L_ at Post‐loss 2, where *C. stellatus* was absent) drives proportionally larger declines (Avolio et al., 2019).

The presence of the Lessepsian invader *B. pharaonis* at all post‐loss sites but not at the control is noteworthy. Although its biomass contribution was modest, consistent with the early stage of its invasion trajectory in the western Mediterranean (Sarà et al., 2013, 2018), its establishment in enriched conditions suggests that organic subsidies from aquaculture may facilitate its settlement by lowering biotic resistance (Table 4). As anthropogenic disturbances intersect with large‐scale biogeographic processes such as Lessepsian migration, these communities may experience delayed but persistent compositional shifts whose functional consequences will unfold over longer time scales (Sarà et al., 2018).

### Environmental correlates and the limits of abiotic monitoring

The correlation analysis between Price components and trophodynamic variables yielded only one association whose bootstrap confidence interval excluded zero (SCE_L_ × TSM, *r* = 0.84), while several others showed large point estimates (e.g., SRE_L_ × CHL‐*a*, *r* = 0.90; CDE × CHL‐*a*, *r* = −0.80) but wide confidence intervals consistent with a sample of only four sites. These results should be interpreted as indicative effect sizes rather than confirmed relationships, and additional data would be needed to test them formally. Nevertheless, the pattern is quite suggestive: Chlorophyll‐*a* and TSM appear associated with components related to species loss and context dependence, whereas the net functional change (Δ*T*) showed weak correlations with all three environmental variables. This decoupling between environmental conditions and net functional outcome is a recurring theme in BEF research (Isbell et al., 2017; Radchuk et al., 2016; Ulrich et al., 2024) and underscores why abiotic monitoring alone may fail to capture the functional consequences of biodiversity change. In the study system, standard water‐quality indicators tracked certain aspects of species turnover but did not predict total biomass change, reinforcing the argument that functional assessment requires direct measurement of biological responses alongside environmental monitoring (Galparsoro et al., 2025).

### Spatial heterogeneity and the need for site‐specific assessment

Despite the consistent overall pattern (positive Δ*T* at every site), the effect size analysis revealed substantial heterogeneity in the magnitude and relative importance of individual components. CDE ranged from a Cohen's *d* of 0.39 (Post‐loss 3) to 1.76 (Post‐loss 1); SCE_G_ varied from −1.41 to −2.69; and the total effect Δ*T* itself spanned a twofold range across sites. This site‐specificity is consistent with simulation studies showing that organic waste deposition from aquaculture facilities is spatially patchy rather than smoothly graded with distance, with local hydrodynamics, substrate type, and bathymetry creating hotspots and gaps within a few hundred meters of the cages (Broch et al., 2017). It follows that sampling designs assuming a uniform gradient of impact may fail to capture critical zones of functional change. Consequently, monitoring frameworks for ecosystem‐based aquaculture should prioritize fine‐scale spatial resolution and tailored site‐specific assessments, moving away from rigid, “one‐size‐fits‐all” geometric grids. Such grids–often predicated on the flawed assumption of isotropic impact–lack ecological grounding and overlook the heterogeneous and patchy nature of benthic responses (Mangano et al., 2023).

### Replicated Price decomposition: A methodological advance

Most applications of the Price equation in BEF research have produced single‐value decompositions for each community comparison, precluding formal uncertainty estimation (Bannar‐Martin et al., 2017; Ladouceur et al., 2022). By exploiting the hierarchical design of the original dataset (multiple CTRL and post‐loss plots), we generated 18 replicated decompositions per site, enabling statistical inference through one‐sample *t* tests and standardized effect sizes. This approach addresses a recognized limitation of the Price framework–namely, the difficulty of assessing whether estimated components differ from zero or from each other (Bourrat et al., 2023; Ulrich et al., 2024). The resulting *p*‐values and Cohen's *d* provide a more rigorous evidence base for interpreting which processes are statistically and ecologically meaningful at each site. We observe, however, that the additive constraint of the Price decomposition (Δ*T* = sum of five components) implies structural nonindependence among components (Ulrich et al., 2024), which should be borne in mind when interpreting inter‐component correlations.

### Interpretive scope, limitations, and paths to validation

The extended Price equation is a mathematical identity that partitions observed functional change into additive components; it diagnoses *where* within the biodiversity‐function relationship the change occurs, but does not identify *why*. Unlike the complementarity‐selection partition of Loreau and Hector (2001), which is anchored to a monoculture null expectation and therefore directly tests the role of interspecific interactions, the Price framework has no built‐in reference for causal inference. In our case, however, the upstream baseline serves as an explicit spatial control against a known environmental gradient, which constrains the range of plausible ecological processes. Table 4 maps each component to the candidate mechanisms most consistent with its observed direction and magnitude, drawing on the known biology of the study system and the literature on fouling community dynamics. These interpretations remain hypotheses, not conclusions. Their validation would require manipulative experiments–most directly, an additive BEF design sensu Loreau and Hector (2001) using monoculture and mixture substrates deployed along the enrichment gradient, which would isolate the contributions of complementarity and selection independently of the Price partition. Similarly, complementary trait‐based approaches (Harrison et al., 2022) could provide further interpretive depth where trait data are available. We view the present results as a diagnostic foundation for such experimental work: The Price decomposition identifies which components carry the largest ecological signal and thereby guides where experimental effort should be directed.

### Implications for ecosystem‐based aquaculture

From a management perspective, our findings bring several practical implications for the EAA. First, the dominant role of CDE indicates that monitoring the *performance* of resident species (as expressed here by their own biomass)–not merely their presence–is critical for detecting functional responses to enrichment. Species‐level biomass time series, rather than species lists or richness indices alone (sensu Gagic et al., 2015), should form the backbone of ecological monitoring at aquaculture sites–a principle that extends to most anthropogenically impacted ecosystems, both marine and terrestrial (Bonilla‐Valencia et al., 2022). Second, the strongly positive SRE_G_ offset by negative SCE_G_ demonstrates that species gains can inflate apparent functional capacity while masking the low individual performance of colonizers; aggregate indicators (e.g., total biomass) may therefore present an overly optimistic picture of ecosystem health. Third, the weak correlation between environmental variables and Δ*T* reinforces the case for integrating biodiversity‐based functional diagnostics alongside standard abiotic monitoring (Brugère et al., 2018; Grebe et al., 2019), as called for by the UNEP/MAP COP23 Decision 26/7 and the EU Biodiversity Strategy 2030 (UNEP/MAP, 2023a, 2023b). Finally, the pronounced site‐specificity of the results argues against one‐size‐fits‐all management zones around aquaculture facilities, supporting instead adaptive, tailored, spatially explicit monitoring that captures local heterogeneity in biodiversity‐function relationships.

## CONCLUSION

This study demonstrates that the extended Price equation, applied to replicated field data, can formally partition the functional consequences of aquaculture‐derived organic enrichment on BEF relationships in Mediterranean fouling communities. The net increase in community biomass at enriched sites was driven by a combination of species gains and enhanced performance of persistent filter‐feeders, partially offset by the low functional contribution of colonizers and the cost of species loss. These patterns are invisible to conventional monitoring based on abiotic variables or aggregate biodiversity indices. By providing replicated, statistically testable estimates of each component, the approach demonstrated here offers a practical and scalable framework for embedding BEF theory into aquaculture impact assessment, contributing to the operationalization of the EAA. Crucially, while the Price decomposition identifies the direction and magnitude of each functional component, it does not test the ecological mechanisms that generate them (Table 4); direct experimental validation–ideally through manipulative BEF designs deployed along enrichment gradients–remains a necessary next step. To our knowledge, this is the first application of the extended Price equation to marine fouling communities under aquaculture enrichment. We encourage its wider adoption in applied marine ecology, coupled with temporal replication and complementary experimental and trait‐based analyses, to build a more process‐informed and management‐relevant understanding of how anthropogenic disturbance shapes ecosystem functioning (Kirkfeldt, 2019).

## AUTHOR CONTRIBUTIONS

Gianluca Sarà, Yun‐Wei Dong and Maria Cristina Mangano conceived the idea and analyzed, wrote, edited, reviewed and funded this ms.

## CONFLICT OF INTEREST STATEMENT

The authors declare no conflicts of interest.

## Supporting information


Appendix S1.

## Data Availability

Data and code (Sara, 2026) are available in Mendeley Data at https://doi.org/10.17632/h9nsr3n88k.1. The original field data derive from Sarà et al. (2007).
