# Supplementary material for: Aquaculture and biodiversity‐ecosystem functioning: A path to lay the foundations of ecosystem‐based approach
Source: Ecol Appl. 2026 Nov 1;36(7):e70334. doi: 10.1002/eap.70334 (PMC13724288; doi:10.1002/eap.70334)
Supplement: Supplementary file 1 [file EAP-36-e70334-s001.pdf]

Appendix S1

Aquaculture and biodiversity-ecosystem functioning: A path to lay the foundations of ecosystem-based approach

Gianluca Sarà, Yun-Wei Dong and Maria Cristina Mangano

Ecological Applications

Section S1: Exclusion criteria for the original post-loss site 3

One of the five post-loss sites originally sampled by Sarà et al. (2007) was excluded from the present analysis. This site (post-loss site 3 in the original numbering of Sarà et al. 2007) was positioned at intermediate distance from the fish cages but exhibited multiple anomalies suggesting that it did not receive effective organic loading from the aquaculture facility. Three independent lines of evidence supported its exclusion:

(1) Community composition. Bray-Curtis dissimilarity between this site and the upstream baseline (CTRL) was 0.20, compared with 0.43-0.70 for all other post-loss sites. This indicates that the community structure was virtually indistinguishable from the undisturbed baseline, inconsistent with a site exposed to organic enrichment.

(2) Particulate organic matter. POM concentration at this site (467 µg l^{-1}) was lower than at the upstream CTRL (516 µg l^{-1}), whereas all other post-loss sites exhibited POM values equal to or higher than the CTRL (570-755 µg l^{-1}). This reversal is consistent with local hydrodynamics deflecting the organic plume away from the site.

(3) Total functional change. This was the only site exhibiting a negative change in total ecosystem function (ΔT = -3.1 g AFDW), whereas all other post-loss sites showed positive ΔT values (+10.8 to +26.0 g). This anomalous negative response, combined with the low Bray-Curtis dissimilarity and below-CTRL POM, indicates that the site did not function as a post-loss comparison site and could not serve as a meaningful data point for the Price equation decomposition.

Exclusion of the original site 3 yielded four retained post-loss sites (renumbered Post-loss 1-4, i.e. PL-1 to PL-4, in the main text) and 72 total pairwise Price decompositions (6 CTRL plots × 3 post-loss plots × 4 sites). We note that the qualitative conclusions are robust to the inclusion of this site: ΔT remains positive at the four retained sites and the pattern of component contributions is unchanged.

Table S1. Plot-level mean ash-free dry weight (AFDW, g) per species across the study area, transposed for readability (species in rows, plots in columns). Values represent the mean of three replicate quadrats (20 × 20 cm) within each plot. Column codes: C1, C2 = baseline CTRL sites; P1-P4 = retained post-loss sites PL-1 to PL-4 (downstream); the number after the dot is the plot (1-3). The original post-loss site 3 (excluded from all analyses, see Section S1) is not shown. Dashes indicate absence.

**Species**	**C1.1**	**C1.2**	**C1.3**	**C2.1**	**C2.2**	**C2.3**	**P1.1**	**P1.2**	**P1.3**	**P2.1**	**P2.2**	**P2.3**	**P3.1**	**P3.2**	**P3.3**	**P4.1**	**P4.2**	**P4.3**
B. perforatus	17.77	5.92	7.81	14.90	0.68	4.48	27.53	27.04	18.64	26.00	18.62	46.14	13.40	14.63	10.43	28.14	22.50	13.08
C. stellatus	1.52	1.95	4.12	15.38	13.22	12.78	0.22	2.17	5.63	–	–	–	2.17	10.83	3.68	0.22	25.57	0.87
B. trigonus	–	–	–	–	–	–	–	–	–	–	–	–	–	–	–	–	3.95	3.64
P. marmoratus	0.12	0.06	0.00	0.00	–	–	0.07	0.05	0.01	0.01	–	0.01	–	–	–	0.06	0.04	–
E. verrucosa	–	–	–	–	–	–	–	–	–	–	–	–	0.01	0.34	0.06	–	0.04	–
A. lunulatus	0.02	–	–	–	–	–	–	–	–	–	0.03	–	–	0.01	0.04	0.05	0.02	–
O. edulis	1.82	2.40	–	0.99	–	33.06	–	–	–	–	–	1.48	–	2.33	3.51	2.20	4.88	1.35
P. coerulea	–	–	–	–	–	0.77	0.59	0.09	0.76	–	0.03	–	–	–	0.26	–	–	–
T. haemastoma	–	–	–	–	–	–	–	0.06	–	–	5.51	–	5.00	–	0.92	–	–	–
A. noae	0.02	–	–	–	–	–	–	–	–	–	–	–	–	–	–	–	–	–
M. minimus	–	–	0.72	0.25	–	0.18	0.88	0.84	0.56	7.76	1.20	3.36	6.48	5.42	17.11	14.15	12.17	11.17
M. galloprovincialis	0.01	–	–	0.15	–	7.26	11.18	0.10	4.24	–	2.02	–	4.06	5.82	–	2.94	–	0.40
M. pharaonis	–	–	–	–	–	–	–	0.10	0.21	0.30	0.07	–	0.10	–	0.81	0.19	0.43	0.49
A. lixula	–	–	–	–	–	–	3.39	2.23	–	6.20	3.69	2.69	4.11	–	–	2.83	–	0.69
**Total**	**21.26**	**10.34**	**12.65**	**31.67**	**13.90**	**58.54**	**43.85**	**32.68**	**30.06**	**40.27**	**31.17**	**53.68**	**35.33**	**39.37**	**36.82**	**50.77**	**69.61**	**31.69**

Table S2. Extended Price equation decomposition of total functional change (ΔT) at each retained post-loss site. Values are mean ± SE across 18 pairwise CTRL-post-loss comparisons per site (6 CTRL plots × 3 post-loss plots). Overall pools all 72 comparisons across the four sites. SRE_L = species richness effect due to loss; SRE_G = species richness effect due to gain; SCE_L = species composition effect due to loss; SCE_G = species composition effect due to gain; CDE = context-dependent effect. Values in g AFDW.

**Component**	**PL-1 (n=18)**	**PL-2 (n=18)**	**PL-3 (n=18)**	**PL-4 (n=18)**	**Overall (n=72)**
**SRE_L**	-4.455 ± 0.958	-12.140 ± 2.247	-6.254 ± 1.220	-4.086 ± 1.242	-6.734 ± 0.828
**SRE_G**	17.399 ± 1.421	22.720 ± 1.673	20.798 ± 1.316	27.073 ± 2.713	21.998 ± 1.002
**SCE_L**	-1.929 ± 2.409	-1.199 ± 2.176	3.595 ± 1.348	3.532 ± 0.931	1.000 ± 0.941
**SCE_G**	-12.019 ± 1.053	-13.993 ± 1.552	-9.801 ± 1.116	-15.213 ± 2.537	-12.756 ± 0.854
**CDE**	11.809 ± 1.584	21.592 ± 2.997	4.111 ± 2.486	14.657 ± 4.769	13.042 ± 1.724
**ΔT**	10.804 ± 4.303	16.981 ± 4.630	12.449 ± 4.071	25.962 ± 5.523	16.549 ± 2.387

Table S3. Standardized effect sizes (Cohen's *d*) for each Price equation component at each retained post-loss site and overall. Cohen's *d* is computed as the ratio of the mean to the standard deviation of the 18 (or 72) pairwise comparisons. Conventional thresholds: $|d| \approx 0.2$ (small), ≈ 0.5 (medium), ≥ 0.8 (large).

Component	PL-1	PL-2	PL-3	PL-4	Overall
SRE_L	-1.096	-1.274	-1.208	-0.775	-0.959
SRE_G	2.886	3.201	3.724	2.352	2.586
SCE_L	-0.189	-0.130	0.629	0.894	0.125
SCE_G	-2.691	-2.125	-2.070	-1.413	-1.760
CDE	1.757	1.698	0.390	0.724	0.892
ΔT	0.592	0.864	0.721	1.108	0.817

References

Sarà, G., M. Lo Martire, G. Buffa, A. M. Mannino, and F. Badalamenti. 2007. The fouling community as an indicator of fish farming impact in Mediterranean. Aquaculture Research 38:66-75. https://doi.org/10.1111/j.1365-2109.2006.01632.x
